# Multiple myeloma with high-risk cytogenetics and its treatment approach

**DOI:** 10.1007/s12185-022-03353-5

**Published:** 2022-05-09

**Authors:** Ichiro Hanamura

**Affiliations:** Division of Hematology, Department of Internal Medicine, Aichi Medical University, 1 Karimata, Yazako, Nagakute, Aichi 480-1195 Japan

**Keywords:** Multiple myeloma, del(17p), t(14;16), gain/amp(1q21), del(1p)

## Abstract

Despite substantial advances in anti-myeloma treatments, early recurrence and death remain an issue in certain subpopulations. Cytogenetic abnormalities (CAs) are the most widely accepted predictors for poor prognosis in multiple myeloma (MM), such as t(4;14), t(14;16), t(14;20), gain/amp(1q21), del(1p), and del(17p). Co-existing high-risk CAs (HRCAs) tend to be associated with an even worse prognosis. Achievement of sustained minimal residual disease (MRD)-negativity has recently emerged as a surrogate for longer survival, regardless of cytogenetic risk. Information from newer clinical trials suggests that extended intensified treatment can help achieve MRD-negativity in patients with HRCAs, which may lead to improved outcomes. Therapy should be considered to include a 3- or 4-drug induction regimen (PI/IMiD/Dex or PI/IMiD/Dex/anti-CD38 antibody), auto-transplantation, and consolidation/maintenance with lenalidomide ± a PI. Results from ongoing clinical trials for enriched high-risk populations will reveal the precise efficacy of the investigated regimens. Genetic abnormalities of MM cells are intrinsic critical factors determining tumor characteristics, which reflect the natural course and drug sensitivity of the disease. This paper reviews the clinicopathological features of genomic abnormalities related to adverse prognosis, focusing on HRCAs that are the most relevant in clinical practice, and outline current optimal therapeutic approaches for newly diagnosed MM with HRCAs.

## Introduction

Multiple myeloma (MM) is a neoplasm of plasma cells that presents with heterogeneous prognostic outcomes. Recent therapeutic advances, such as the introduction of immunomodulatory drugs (IMiDs), proteosome inhibitors (PIs), and anti-CD38 antibodies, have greatly improved outcomes in patients with newly diagnosed (ND) MM [1]. However, around 10–20% of patients still experience early death within 2–3 years of diagnosis; these cases are usually defined as high-risk MM [1, 2].

Parameters predicting poor outcomes are well established, including cytogenetic abnormalities (CAs) such as t(4;14), t(14;16), t(14;20), ≥ 3 copies of chromosome band 1q21 (1q21 +), deletion of chromosome arm 1p (del(1p)), and deletion of chromosome band 17p13 (del(17p)) (Table 1); clinical biomarkers and features such as ISS stage 3 [3], high serum lactate dehydrogenase (LDH) [4], the presence of circulating plasma cells including plasma cell leukemia (PCL) [5], central nervous system (CNS) involvement [6], and plasmablastic morphology [7]; and host factors such as age, renal dysfunction, and frailty [8]. Other molecular parameters can also predict outcomes (Table1), such as gene expression profiling (GEP) signatures, including the GEP70-gene signature from University of Arkansas for Medical Sciences (UAMS) [9] and the EMC-92 signature from the HOVON group [10]; a gene mutational status such as APOBEC (apolipoprotein B mRNA-editing enzyme, catalytic polypeptide)-type mutational signatures [11]; a high total number of mutations [12]; the presence of homologous recombination deficiency [13]; and the presence of chromothripsis [14, 15]. In addition to predictors at presentation, the minimal residual disease (MRD) status has emerged as a strong indicator of prognosis in patients with NDMM. Durable MRD-negativity has become a surrogate for longer progression-free survival (PFS) and overall survival (OS), regardless of cytogenetic risk [16, 17]. Many clinical trials are currently being conducted using achievement of MRD-negativity as a primary endpoint, which will provide insight into treatment strategies for NDMM patients with high-risk features in the short term. Besides tumor cells, recent studies have suggested that the immune status of the tumor microenvironment is likely to play a pathogenic role in MM development and drug efficacy [18].

**Table 1:** Adverse cytogenetic and molecular abnormalities

Cytogenetic abnormalities	%, approximate
t(4;14)	15
t(14;16)	4
t(14;20)	2
del(17p)	5–10
del(1p)	20–30
1q21 +	30–40

Molecular abnormalities	%, approximate
*TP53* mutations	5
APOBEC signature	− 15
Chromothripsis	− 20
Homologous recombination deficiency	3
GEP signature (UAMS70, and SKY92)	15–20

*APOBEC* apolipoprotein B mRNA-editing enzyme, catalytic polypeptide, *GEP* gene expression profiling, *UAMS* University of Arkansas for Medical Sciences

Thus, there are many factors affecting prognosis in MM, many of which overlap. However, CAs detected by fluorescence in situ hybridization (FISH) are currently the most widely available and accepted as prognostic markers in our daily clinical practice. Since post-relapse treatment for high-risk patients is usually ineffective [19], recognizing the risk profile in NDMM patients and maintaining remission in patients with high-risk features are clinically important for their longer survival [20]. This paper reviews the clinicopathological features of genomic abnormalities that are related to adverse prognosis, with a focus on high-risk (HR) CAs, and outlines current optimal therapeutic approaches for NDMM patients with HRCAs by reviewing recent clinical trials.

## Pathogenic role of cytogenetic abnormalities in MM

### Cytogenetic abnormalities related to myelomagenesis

MM develops through a multistep process involving genomic instability during which clonal evolution occurs [21–23]. Genomic abnormalities observed in almost 100% of clonal MM cells of patients present at diagnosis are considered to be related to disease initiation, and those observed in subclones of the tumor are considered secondary events [21]. From this viewpoint, MM is thought to initiate via hyperdiploid (HDR) or chromosomal translocations involving an immunoglobulin heavy chain gene (*IGH*) locus [24, 25]. Chromosomal copy number abnormalities (CNAs) and somatic gene mutations are considered secondary events associated with disease progression [22] [23].

HDR is usually defined by the number of chromosomes ≥ 50 in the tumor cells. In MM, HDR is characterized by simultaneous trisomy of chromosomes 3, 5, 7, 9, 11, 15, 19, and 21 [24, 25]. HDR is a better prognostic marker in patients with NDMM [26]. In patients with HDR, those with gain/amp(1q21), del(17p), diploid of chromosome 11, or trisomy of chromosome 21 have been reported to show poor prognosis compared with patients lacking these abnormalities [27–30].

Non-HDR MM is characterized by *IGH* translocations [24, 25]. HDR and *IGH* translocations are usually mutually exclusive events in myelomagenesis [24, 25]. In the process of B-cell differentiation, the *IGH* region undergoes V(D)J recombination, receptor revision, somatic hypermutation, and class switch recombination; thus, the *IGH* locus is genetically “unstable”, and *IGH* translocations are frequently found in B-cell malignancies [31]. In plasma cells, the activity of the *IGH* enhancer is extremely high, so that large amounts of immunoglobulin can be translated [21]. As a result of *IGH* translocations, oncogenes translocated near the *IGH* enhancer are strongly expressed [21, 32]. Primary *IGH* translocations associated with myelomagenesis have breakpoints in the region related to B-cell differentiation, while secondary *IGH* translocations associated with disease progression, after the tumor transformation of plasma cells, have breakpoints in other regions [21]. The primary *IGH* translocations are t(11;14), t(6;14), t(4;14), t(14;16), and t(14;20), leading to the ectopic overexpression of *CCND1, CCND3, MMSET/FGFR3, MAF,* and *MAFB*, respectively [33–37]. MAFA, a member of the large MAF family, is rarely overexpressed following *IGH* translocations [38]. *MAFA* is located at chromosome band 8q24, about 16 Mb on the telomere side of *MYC*, which might be undistinguishable from *MYC* by G-band cytogenetics. *CCND2*, a member of the CCND family, is also rarely overexpressed by the *IGH* translocation t(12;14) [38]. All primary *IGH* translocations induce the overexpression of either *CCND1-3*; *CCND1* by t(11;14), *CCND2* by t(12;14), t(4;14), t(14;16), t(14;20), or t(8;14) (*IGH-MAFA*), and *CCND3* by t(6;14) [38–40]. In patients with HDR, patients with the trisomy of chromosome 11 show a tendency for overexpressed *CCND1*, and those with the diploid of chromosome 11 does for *CCND2* [29]. Dysregulation of *CCND* is thought to be essential for myelomagenesis. Sequence analysis of surrounding breakpoints in the *IGH* loci in patient samples by next-generation sequencing (NGS) have indicated that *IGH* translocations occur predominantly in germinal center B-cells, and also occur in early pro-B-cells in around 20% of patients with t(11;14) and t(14;20) [41]. This suggests that determination of differentiation into plasma cells of B-cells is initiated before the early pro-B-cell stage. The clonal origin of MM cells in a patient might be related with tumor characteristics, which may affect the natural disease course and drug sensitivity; however, the association has not been investigated.

### High-risk cytogenetic abnormalities in MM

Prognostic indicators change over time with advances in therapeutic agents and newly identified indicators. Several CAs have been shown to be associated with poor prognosis, even in the era of novel drugs, such as t(4;14), t(14;16), t(14;20), 1q21 + , del(1p), and del(17p) [42–45]. Interphase FISH is useful for detecting already known chromosomal abnormalities in patient MM cells, because fresh MM cells are usually difficult to grow in vitro due to a lack of cytokines and interactions with bone marrow stroma [46]. Capturing abnormal G-band images of patient MM cells indicates that those cells can grow without marrow support in the short term at least. Co-existence of unfavorable CAs has been suggested to indicate an even worse prognosis than a single abnormality [45, 47, 48]. In a similar context, adverse impacts of t(4;14) alone [49, 50] and gain(1q21) (three copies of chromosome arm 1q21) alone are likely to be overcome [51, 52]. Recently, the MASTER clinical trial showed that Dara-KRd + ASCT + Dara-KRd could abrogate the adverse effects of isolated HRCA, including t(4;14), t(14;16), 1q21 + , and del(17p), in transplant-eligible NDMM [45].

#### Primary cytogenetic events related to adverse outcomes

##### t(4;14)(p16;q32)

The translocation t(4;14)(p16;q32) is found in around 15% of NDMM. In all patients with this translocation, an aberrant IGH-MMSET transcript is formed, and *MMSET* is activated by the *IGH* enhancer μ [35]. *FGFR3*, a tyrosine kinase receptor, is located about 60 kb on the centromeric side of *MMSET* and is overexpressed in the vicinity of the *IGH* 3ʹ enhancer α. The overexpression of *FGFR3* is not observed in about 30% of patients with t(4;14) due to deletion of der[14] carrying *FGFR3* [53, 54]. MMSET is a histone methyltransferase that alters the histone methylation status in the entire genome and mediates gene-specific DNA hypermethylation [55]. Martinez-Garcia et al. reported that overexpression of MMSET is correlated with an increase in lysine 36 methylation of histone H3 (H3K36me2) and a decrease in lysine 27 methylation of histone H3 (H3K27me2) across the genome in MM cells, which affects cell adhesion, growth, and survival [56]. Krijger et al. reported that MMSET promotes non-homologous end-joining (NHEJ) at deprotected telomeres in MM cells [57], suggesting that MMSET may affect the DNA repair process. However, the specific mechanisms associated with poor prognosis resulting from t(4;14) are largely unknown.

##### t(14;16)(q32;q23) and t(14;20)(q32;q11)

t(14;16)(q32;q23) and t(14;20)(q32;q11) are observed in around 4% and 2% of NDMM patients in which *MAF* and *MAFB* are overexpressed, respectively. MAF and MAFB are transcription factors, members of the large MAF family, and function by binding to the MARE (MAF recognition element) sequence in the promoter region of various target genes. Ectopic overexpression of large MAFs results in dysregulated expression of downstream genes, such as *CCND2, ARK5, integrin β7* (*ITGB7)*, and *APOBECs* [11, 40, 58]. Translocations of large MAF genes to *IGH* loci are found infrequently at diagnosis; however, their prognosis is sometimes dismal, so elucidation of the biology resulting from these translocations is necessary to improve outcomes of MM.

ARK5, adenosine monophosphate (AMP)-activated protein kinase-related kinase 5 (also known as NUAK1, NUAK family, SNF1-like kinase 1), was initially reported by Suzuki A. et al. as a novel AMPK family member and a tumor survival factor under nutrient starvation, which is activated by Akt and functions as an ATM kinase [59]. ARK5 is upregulated by MAF and MAFB through the MARE sequence in MM cells [58]. In several cancers, overexpression of *ARK5* is related to tumor invasion, metastasis, and poor prognosis, with downregulation of *ARK5* resulting in improved sensitivity to anti-tumor drugs [60]; thus, ARK5 is a potential therapeutic target in MM with t(14;16) or t(14;20).

ITGB7 is an integrin protein that is associated with cell adhesion, migration, and invasion [61]. In MM cells, overexpression of *ITGB7* enhances these functions, which are related with cell-adhesion-mediated drug resistance [62]. MMG49, chimeric antigen receptor T cells (CAR-T cells) targeting the activated ITGB7 protein are a potential therapeutic option for patients with t(14;16) or t(14;20) [63, 64].

APOBEC3A, APOBEC3B, and APOBEC4 are overexpressed in patients with t(14;16), and APOBEC4 is overexpressed in those with t(14;20) [11]. The APOBEC family is a group of enzymes that has the ability to convert cytosine to uracil in DNA/RNA. APOBEC3G, a member of the APOBECs, was originally reported as a protein that introduces mutations into the viral genome of human immunodeficiency virus (HIV), resulting in inhibition of viral replication [65]. The analysis of NGS data from databases of various human cancers has revealed that APOBEC3-type mutational signatures (APOBEC signatures), in which cytidine is frequently converted to uracil/thymidine in TCN trinucleotide repeats, are present in 16 of 30 cancers [66]. The APOBEC signature has been observed in about 20% of all mutations in cancer cells, which is the second highest frequency after the age-related mutational signature in all types of cancers [66]. In MM, the APOBEC signature is associated with a higher total number of mutations in the entire genome and poor prognosis [11, 12]. The total number of genomic mutations is highest in the t(14;16) group among subgroups classified by primary *IGH* translocations, HDR, and major secondary CAs [11, 12]. Elevated expression of APOBEC is thought to induce gene mutations and chromosomal instability over time. The negative prognostic impacts of t(14;16) and t(14;20) may be due, in part, to the high proportion of patients with the APOBEC signature in these populations.

Overexpression of transcription factors, large MAFs, and the histone methyltransferase MMSET induces altered expression of a wide variety of associated genes. Therefore, the mechanisms of tumor malignancy are multifaceted and complicated by *IGH* translocations with one gene of the large MAFs or *MMSET*, which may also in part account for the poor prognosis in patients with these translocations.

#### Secondary cytogenetic events related to adverse prognosis

CAs other than HDR and the primary *IGH* translocations are considered secondary events, including CNAs and chromosomal translocations. Secondary chromosomal events might occur randomly resulting from chromosomal instability of MM cells, but several seem to have pathological significance. Currently, the most widely accepted adverse CNAs are 1q21 + , del(1p), and del(17p) [1]. Secondary abnormalities, in combination with primary and/or other secondary abnormalities, can form more resistant clones [45], although the synergistic molecular pathological effects resulting from the co-occurrence of HRCAs are not well characterized.

## Gain/amplification of chromosome arm 1q21 (1q21 +)

Gain/amplification of chromosome arm 1q21 (1q21 +) is observed in around 40% of patients at diagnosis [67]. The incidence of patients with 1q21 + increases from monoclonal gammopathy of undetermined significance (MGUS) (0–20%) to refractory/relapse (RR) MM (≥ 50%) [67, 68]. 1q21 + is likely to be linked to a higher risk for progression from smoldering MM to MM [67, 69]. These observations suggest that 1q21 + is associated with disease progression and drug resistance. 1q21 + can be divided into two groups according to the increased levels of 1q21 copy numbers, either gain(1q21) (3 copies of 1q21) or amp(1q21) (≥ 4 copies of 1q21). The prognosis is worse in patients with amplification than in those with gain [70], and recent studies suggest that the adverse effects of gain(1q21) can be abrogated by carfilzomib-based treatment in the FORTE trial [52].

In studies analyzing metaphase spreads from MM patient samples, 1q21 + is likely caused by chromosomal instability of 1q12, resulting in jumping translocation of the whole chromosome arm 1q to other chromosomes (JT1q), and segmental duplications of 1q12-25 (dup(1q21)) [71, 72]. JT1q can induce arm-level and/or partial losses of associated receptor chromosomes, in addition to gain of the whole chromosome arm 1q. JT1q may enhance chromosomal instability that results in chromosomal losses in MM cells because the associated receptor chromosomes can be ‘unstable’ during mitosis. Several genes situated in the 1q21 amplicon are also likely to confer the adverse effects of 1q21 + , such as *ANP32E, MCL1, PSMD4, ILF2, IL6R, ADAR, CKS1B*, and *PBX1,* especially in cases of dup(1q21) [73]. The simultaneous enhanced function of the expressed genes of the 1q21 amplicon may affect the resistance of different drugs. Recent studies have indicated that MM cells with 1q21 + seem to be more sensitive to inhibitory agents of MCL1 [74] and the PBX1-FOXM1 axis [75] compared with those lacking 1q21 + .

1q21 + cells constantly increase the copy number of 1q21 over time [67], and patients with amp(1q21) show worse prognosis compared with those without amp(1q21), even in relapsed patients [67, 76]. Therefore, eradication of 1q21 + cells early in the treatment may be important to improve the outcome of MM patients with 1q21 + .

## Deletion of chromosome arm 1p

About 30% of NDMM patients have deletion loci somewhere in chromosome arm 1p [77]. Most patients with del(1p) have internal deletions, and around 15% have deletion of the whole arm of 1p [77]. Del(1p) has been shown to be associated with poor prognosis for MM [26, 78]. The candidate genes of del(1p) have been suggested as *CDKN2C* and *FAF1* at 1p32, *RPL5* and *EVI5* at 1p22, and *FAM46C* at 1p12 [77]. CDKN2C inhibits the cell cycle; therefore, loss of CDKN2C function is likely to confer the enhancement of cell proliferation, although *CCND* dysregulation has already occurred in MM cells. *FAF1* is a gene associated with the induction of apoptosis, so the loss of FAF1 seems to result in anti-apoptosis in cells. *FAM46C* mutations are found in around 8% of patients at diagnosis [77, 79]. Of the *FAM46C* mutations, around 60% are single nucleotide variations, 20% are frameshift indels, and the remaining 20% are in-frame indels. About 40% of *FAM46C* mutations in myeloma cells are between codons 173 and 186, suggesting that this may constitute a mutation hotspot [78]. The function of *FAM46C* in myeloma cells is not completely elucidated, but it has been reported to function as a non-canonical poly (A) polymerase, which can stabilize mRNA [80, 81] and act as a tumor suppressor [82]. More recently, FAM46C has been reported to form a complex with FNDC3A, which is involved in the endoplasmic reticulum stress response and regulation of autophagy [83]. We have also reported that bi-allelic deletion of *FAM46C* by the CRISPR-Cas9 system enhances cell proliferation along with activation of PI3K-Akt signaling, and the Akt inhibitor afuresertib is more effective in suppressing cell growth in MM cells with disrupted *FAM46C* than in parent cells with wild-type (WT) *FAM46C* [84]. A phase 1 clinical trial investigating single agent afuresertib in RRMM (NCT 00881946) demonstrated a favorable safety profile, showing clinical effectiveness of afuresertib in subpopulations [85], but clinical trials of afuresertib for MM are not currently being conducted. Patients with disruption of *FAM46C* might be good candidates for clinical trials of afuresertib.

## Deletion of chromosome arm 17p (del(17p))

Del(17p) is observed in 5–10% of patients at diagnosis, and has been associated with poor prognosis in MM [26, 70, 86, 87]. *TP53*, located on chromosome band 17p13, is assumed to be the responsible gene of del(17p) in MM. The deletion and mutations of *TP53* are found in around 9% and 5% of NDMM patients, respectively [29]. Biallelic events (deletion + mutation) of *TP53* are found in around 4% of NDMM patients [29], which indicates that the mutations of *TP53* are enriched in clones with del(17p) in MM. Mutation sites are predominant in the DNA-binding domain (around 80%) in NDMM patients [29].

p53, encoded by *TP53*, plays a critical role in maintaining genomic integrity and cellular homeostasis, which prevents tumorigenesis and cancer cells from becoming more malignant [88]. Activation of p53 is triggered in response to numerous endogenous and exogenous cellular stresses, such as hypoxia, replication stress, oncogenic activation, nutrient starvation, and cytotoxic agents. p53 exerts its effects via transcription-dependent manners and/or direct protein–protein interactions, and participates in a complicated signaling network involved in regulating cell cycle arrest, senescence, apoptosis, and metabolism [88].

In MM, del(17p) is the most notorious adverse prognostic marker, and bi-allelic inactivation of *TP53* shows the worst prognosis among patients with del(17p) [70]. Some p53 mutants show oncogenic properties [88]; however, in MM, p53 mutants are thought to work as tumor suppressors [29]. In other respects, it is interesting that the cancer clonal fraction (CCF) of del(17p) affects the prognosis of MM [87]. The prognosis is poorer when the CCF of del(17p) cells is about 60% or more in all myeloma cells in patients; however, the prognosis is not significantly affected below this level [68]. It has been reported that *TP53* mutations are enriched in populations with high CCF of del(17p), which may be part of the reason for the poorer prognosis in patients with higher CCF of del(17p) [89].

Regarding treatment strategies for patients with del(17p), MM cells should be eradicated while the cells have at least one WT *TP53*, because WT *TP53* might disappear at relapse [90]. Enhancement of the function of residual WT p53 via downregulation of p53-inhibiting molecules, such as MDM2 [91], might be beneficial for patients with haploinsufficiency of *TP53*. Clarification of the cellular pathways inducing p53-independent cell death may assist in the development of treatment strategies for patients with null WT *TP53,* since restoration of WT *TP53* is not possible.

Tandem ASCT incorporating bortezomib appears to overcome the adverse effect of del(17p) in subpopulations [92, 93].

## Effects of gene mutations on prognosis and the targeted therapies

In a study by Walker et al., in which the pooled whole-exon sequencing data of 1273 patients with NDMM were analyzed, there were over 60 mutated genes in patients [29]. Frequently mutated genes were *NRAS* (21%), *KRAS* (17%), *DIS3* (9%), *FAM46C* (8%), *BRAF* (7%), and *TP53* (5%) and most gene mutations were less recurrent. The gene mutation associated with adverse OS by multivariate analysis was only the *TP53* mutation [70].

*RAS* mutations are the most frequently observed in patients with NDMM [29, 94], and are associated with the progression from smoldering MM to MM. *RAS* mutations are not associated with poor prognosis in NDMM patients, which is explained, in part, by *N/K-RAS* mutations being found more frequently in patients with HDR. Sotorasib, a direct inhibitor of the KRAS G12C mutant, was approved by the FDA for non-small cell lung cancer with this mutation [95], and it may have a benefit for the treatment of MM patients with this mutation. However, inhibiting the RAS signaling network might be a better strategy [96], compared with developing all direct inhibitors for each *RAS* mutation because there are many types of the *RAS* mutations in MM.

Recent detailed genomic analysis of cancer cells using NGS has established the concept of genomic heterogeneity and the clonal evolution similar to Darwin's theory in cancers, resulting in drug resistance. In MM, therapeutics targeting specific mutations are unlikely to succeed due to intra-patient and intra-clonal genetic heterogeneity and the co-occurrence of multiple genetic abnormalities in MM cells. In addition, if one specific pathway is suppressed, another pathway may be activated, which can help the cell survive. Effective combination therapies of targeted drugs, along with the elucidation of important pathways associated with aggressiveness and/or drug resistance, may open up the potential for targeted therapies in MM.

## GEP associated with poor prognosis

Gene expression status, defined by using GEP, can help with risk stratification of MM; these include the GEP70-gene signature (UAMS70) [9] and EMC92-gene signature (SKY92) [10]. UAMS70 and SKY92 were both developed based on GEP with the Affymetrix gene chip U133 plus2.0. UAMS70 and SKY92 were developed to predict patients with early disease-related deaths that constituted around 15% of those in the Total Therapy 2 and 3 trials at UAMS, and with an OS of less than two years in the HOVON-65/GMMG-HD4 trial, respectively. Of the 70 genes in UAMS70, 9 of the 51 upregulated genes mapped to chromosome 1q, and 9 of the 19 downregulated genes mapped to chromosome 1p, suggesting that UAMS70 is relevant to a subtype with both 1q21 + and del(1p) [9]. UAMS70 and SKY92 have similar trends, with higher incidences of del(13q), del(17p), 1q21 + , IGH split, t(4;14), and t(14;16) in the high-risk group, and higher incidences of HDR in the standard-risk group, while there are only two genes common to both models [10].

## International prognostic classification systems for NDMM

The prognostic classification systems for MM include ISS, R-ISS, and the recently proposed R2-ISS [97]. ISS was established in the 1990s prior to the introduction of novel drugs such as IMiDs and PIs; however, at present, ISS stage 3 is usually correlated with poor prognosis [98]. ISS is a simple staging system that is divided into three groups using only two parameters: serum albumin and β2 microglobulin (β2MG) levels. β2MG is a single-chain polypeptide that is bound to the heavy chain of the HLA class I molecule as the light chain. β2MG is expressed on almost all cell surfaces except erythrocytes, and is especially abundant in lymphocytes and monocytes. This low molecular weight polypeptide passes through the glomerular basement membrane and is mostly reabsorbed in the renal tubules. High serum β2MG levels reflect both high tumor load and renal damage in patients. The specific biological reason why hypoalbuminemia is related to poor prognosis is unclear in MM, but ISS stage 3 represents the highest risk. To more clearly stratify patients with worse and better prognoses, R-ISS (revised ISS) has been established, which is a system that combines ISS with HRCAs, including t(4;14), t(14;16), del(17p), and very high LDH, but does not include 1q21 + [99]. In a study by Walker et al. of 784 patients having ISS, age, PFS, OS, and genomic data were analyzed, and the highest-risk patients included either bi-allelic inactivation of *TP53* or ISS stage 3 with amp(1q21), which represented around 6% of all patients [70]. R2-ISS is a system recently proposed by the European Myeloma Network (EMN), and includes 1q21 + as a parameter [100]. R2-ISS is divided into four groups according to the total score using the parameters ISS stage 2 (1 point), ISS stage 3 (1.5 point), del(17p) (1 point), very high LDH (1 point), t(4;14) (1 point), and 1q21 + (0.5 point). t(14;16) is detected in around 5% of NDMM and is usually considered a HRCA; however, it was omitted as a parameter of R2-ISS because it was correlated with OS but not PFS [100]. Infrequent abnormalities, such as t(14;16) and most somatic gene mutations could be underrepresented in statistical prognostic analysis.

## Prognostic significance of MRD-negativity in patients with HRCAs

**Fig. 1:**
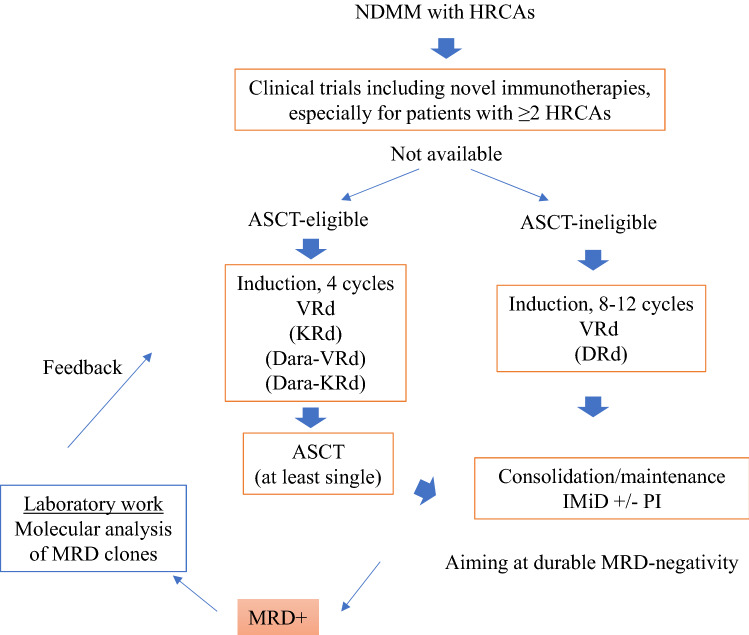
Treatment approach for patients with newly diagnosed multiple myeloma and high-risk cytogenetic abnormalities. *MM* multiple myeloma, *ASCT* autologous stem cell transplant, *d* dexamethasone, *Dara* daratumumab, FISH fluorescent in situ hybridization, *K* carfilzomib, *R* lenalidomide, *V* bortezomib, *HRCA* high-risk cytogenetic abnormality, *MRD* minimal residual disease

MRD is a high-sensitive measure of residual MM cells in bone marrow, which can currently be evaluated using with multicolor flow cytometry or NGS to detect MRD sensitively up to around 10^–6^ of all bone marrow cells [101]. MRD-negativity is associated with improved survival outcomes in MM, regardless of MRD assessment methods or cytogenetic risk [16, 102, 103]. In addition, sustained MRD-negativity is becoming a surrogate for PFS and OS [104]. In patients who achieve MRD-negativity but fail it after a short time, the MM cells may remain at sub-sensitivity levels or somewhere in the patients` body other than at the aspiration site of the bone marrow, and later become active above threshold levels. In addition to the clinical use of MRD, characterization of MRD cells, which can be sorted by FACS, may provide clues for developing the novel therapeutic strategies for refractory MM (Fig. 1). In a study by Gicoechea et al. [105], in which the paired tumor cells at diagnosis and the MRD level from the PETHEMA/ GEM2012 MENOS65 trial were analyzed with whole-exon and RNA sequencing, MRD cells displayed reactive oxygen species (ROS)-mediated drug resistance in patients with HRCAs, while those did greater clonal selection pattern in patients with standard-risk CAs [105].

## Treatment approach for NDMM with HRCAs

Patients with HRCAs are more prone to poor prognosis than those lacking HRCAs; however, the most important issue is whether those patients should be treated in a different manner using currently approved anti-myeloma drugs. Clear evidence based on randomized trials in enriched patients with HRCAs is lacking, other than the SWOG 1211 trial, but high-risk subgroup analysis and other recent clinical studies (Table 2) suggest that treatment for patients with HRCAs should include a three-drug regimen of PI/IMiD/Dex (or + an anti-CD38 antibody if possible), high-dose chemotherapy with ASCT if eligible, and post-ASCT therapy including consolidation and maintenance, aiming at MRD-negativity (Fig. 1).

**Table 2:** Selected clinical trials and high-risk subgroup analysis in patients with newly diagnosed multiple myeloma

Trial	Total patient number	Regimen	High-risk subgroup				
			Definition^a^		%, among evaluable patients	PFS hazard ratio (95% confidence interval) and/or MRD-negativity rates	References
Transplant ineligible or not intent immediate ASCT							
SWOG S0777 Phase 3	471	VRd vs Rd	del(17p), t(4;14), t(14;16)		Analyzed 44 patients (8% in all cohort)	Median PFS 38 vs 16 months (*p *= 0.19)	[106]
MAIA Phase 3	737	Dara-Rd vs Rd	del(17p), t(4;14), t(14;16)		12%	0.85 (0.44–1.6)	[110]
ALCYONE Phase 3	706	Dara-VMP vs VMP	del(17p), t(4;14), t(14;16)		14%	0.78 (0.43–1.4)	[112]
SWOG 1211 Phase 2	100	Elo-VRd vs VRd	del(17p), t(4;14), t(14;16), t(14;20), 1q21 + , PCL, UAMS70, high LDH		100%	0.96 (80% confidence interval 0.69–1.3)	[108]
TOURMALINE-MM2 Phase 3	705	IRd vs Rd	del(17p), t(4;14), t(14;16), 1q21 +		39%	0.69 (0.51–0.94)	[117]
ENDURACE Phase 3	1087	KRd vs VRd	All without del(17p), t(14;16), t(14;20)		t(4;14), 7%	1.1 (0.54–2.4)	[109]
Transplant eligible							
IFM2009/DFCI Phase 3	700	VRd + ASCT vs VRd	del(17p), t(4;14), t(14;16)		17%	0.90 (not significant)	[118]
CASSIOPEIA Phase 3	1085	Dara-VTd + ASCT vs VTd + ASCT	del(17p), t(4;14)		15%	0.67 (0.35–1.3) MRD-negativity (10^–5^) at day100 ASCT, 59% vs 44%, odds ratio 1.8 (1.02–3.4)	[113]
GRIFFIN Phase 2	207	Dara-VRd + ASCT vs VRd + ASCT	del(17p), t(4;14), t(14;16)		15%	MRD-negativity (10^–5^) at the end of consolidation, 37% vs 28%, odds ratio 1.5 (0.32–6.9)	[121]
FORTE Phase 2	474	KRd + ASCT vs KRd vs KCd + ASCT	del(17p), t(4;14), t(14;16), 1q21 +		54%	KRd-ASCT vs KRd alone, 0.48 (0.27–0.86)	[122]
						KRd-ASCT vs KCd-ASCT, 0.50 (0.28–0.89)	
MASTER Phase 2, single arm	123	Dara-KRd + ASCT + Dara-KRd (MRD guided)	del(17p), t(4;14), t(14;16), t(14;20), 1q21 +		57%	MRD negativity (10^–5^), 78%, 82%, and 79% for patients with 0, 1, and > = 2 HRCAs, respectively	[45]
						Two-year PFS, 91%, 97%, and 58% for patients with 0, 1, and > = 2 HRCAs, respectively	
OPTIMUM Phase 2, single arm	107	Dara-CVRd + ASCT^b^ + Dara-VR(d)	At least 2 high-risk cytogenetics (t(4;14), t(14;16), t(14;20), 1q21 + . del(1p), del(17p)), SKY92, PCL		100%	MRD-negativity (10^–5^), around 50% and 80% at post induction and day100 post ASCT, respectively (% of MRD-evaluated patients)	[123]

*MM* multiple myeloma, *PFS* progression free survival, ASCT Autologous stem cell transplant, *C* cyclophosphamide, *d* dexamethasone; *Dara* daratumumab, *FISH *fluorescent in situ hybridization, *K* carfilzomib, *M* melphalan, *P* prednisone, *R* lenalidomide, *T* thalidomide; *V* bortezomib, *UAMS* University of Arkansas for Medical Sciences, *HRCA* high-risk cytogenetic abnormality, *PCL* plasma cell leukemia, *MRD* minimal residual disease

^a^The cytogenetic abnormalities were defined by fluorescence in situ hybridization (FISH) or cytogenetics or others. Cutoff points for presence of abonormal FISH are different among studies

^b^High-dose melphalan augmented with bortezomib

### Patients with HRCAs, without ASCT

The phase 3 SWOG S0777 study established bortezomib, lenalidomide, and dexamethasone (VRd) as the standard-of-care for transplant-ineligible NDMM patients [98, 106, 107] (Table 2). The S0777 study compared VRd to Rd in NDMM without an intent for immediate ASCT [98, 106]. Patients were assigned to 8 × VRd or 8 × Rd, followed by Rd maintenance. VRd showed superior PFS and OS compared with Rd (VRd vs. Rd, medium PFS/OS, months: 40/not reached (NR) vs. 28/60, *p *= 0.003/0.011, respectively). Regarding the effects of VRd for patients with HRCAs, in patients with t(4;14) and/or del(17p), PFS and OS in the VRd group showed superior trends compared with those in the Rd group, though without statistical significance. This may be due to insufficient cytogenetic data to establish efficacy in patients with HRCAs.

Usmani et al. first reported a randomized phase 2 trial (SWOG 1211) for enriched NDMM patients with high-risk features, comparing elotuzumab and VRd (Elo-VRd) vs. VRd alone [108] (Table 2). High-risk features included UAMS70 high-risk signature, t(14;16), t(14;20), del(17p), 1q21 + , primary plasma cell leukemia, and elevated serum LDH (≥ two times the upper limit of normal). Elo-VRd and VRd were continued until disease progression. Unfortunately, the addition of elotuzumab to the induction and maintenance phases did not improve outcomes compared with VRd alone (medium PFS, Elo-VRd vs. VRd: 31 months vs. 34 months, respectively, which was not statistically significant). PFS in both groups exceeded statistical estimates, suggesting that continuous maintenance therapy with the PI-IMiD combination may be beneficial for patients with high-risk features.

The phase 3 ENDURACE study compared carfilzomib, lenalidomide, and dexamethasone (KRd) to VRd in NDMM patients with standard-risk CAs and t(4;14), who were not being considered for immediate ASCT [109] (Table 2). Patients were assigned to induction therapy with VRd or KRd for 36 weeks, and were subsequently assigned to either indefinite maintenance or two-year lenalidomide maintenance. KRd did not improve PFS compared with VRd in the overall cohort (medium PFS, KRd vs. VRd: 34 months vs. 34 months, respectively, *p *= 0.74) or in the subpopulation with t(4;14). This suggests that KRd cannot improve outcomes of patients with t(4;14) compared with VRd.

Daratumumab (Dara), lenalidomide, and dexamethasone (DRd) is also the standard-of-care regimen for transplant-ineligible NDMM. The phase 3 MAIA study showed that the addition of Dara to Rd significantly improved PFS and MRD-negativity rates [110] (Table 2). With longer follow-up, DRd maintained a PFS benefit and deeper and more durable responses compared with Rd [111]. DRd was more effective for patients with standard-risk CAs (hazard ratio 0.5, 95% CI 0.38–0.65) compared with those with HRCAs (hazard ratio 0.57, 95% CI 0.32–1.03) [110]. The phase 3 ALCYONE trial showed that the addition of Dara to VMP (bortezomib + melphalan + prednisone) significantly improved PFS compared with VMP alone for transplant-ineligible NDMM, but the PFS benefit of Dara-VMP was not seen in patients with HRCAs [112] (Table 2). A pooled meta-analysis including MAIA, ALCYONE, and CASSIOPEA (Dara-VTD vs. VTD for transplant-eligible NDMM) [113–115] demonstrated that the addition of Dara to each control arm at induction was associated with superior PFS (hazard ratio 0.67, 95% CI 0.47–0.95) in patients with HRCAs. The phase 3 CEPHEUS trial, comparing the efficacy of Dara-VRd and VRd for transplant-ineligible NDMM patients, will provide the value of the addition of Dara to VRd for this patient population, including high-risk patients [116].

The phase 3 TOUMALINE-MM2 study, comparing ixazomib + Rd (IRd) and Rd in transplant-ineligible NDMM patients, did not show statistically superior PFS in the IRd group compared with the Rd group (median PFS, 35 months vs. 22 months; hazard ratio, 0.83; *p *= 0.07) [117] (Table 2). However, PFS with IRd was better than that with Rd in patients with HRCAs, which included t(14;16), t(14;16), del(17p), and 1q21 + (median PFS, 23 months vs. 18 months; hazard ratio 0.69; *p *= 0.019).

In summary, the addition of bortezomib, ixazomib, or Dara to Rd, compared with Rd alone, potentially improves PFS in transplant-ineligible NDMM patients with HRCAs. Continuous VRd also may help improve outcomes in this population.

### Patients with HRCAs and ASCT

The phase 3 IFM2009/DFCI study compared VRd + ASCT to VRd alone in transplant-eligible NDMM. Patients were assigned to 3 × VRd + ASCT + 2 × VRd, or 8 × VRd alone, followed by 1-year lenalidomide maintenance. VRd + ASCT showed significantly longer PFS than VRd alone, but OS was similar between the groups [118] (Table 2). The PFS benefit of ASCT was seen in patients with standard-risk CA, but not in those with HRCAs. MRD negativity (10^–6^) was achieved at least once during maintenance in 17 of 42 patients with t(4;14) (40%), but in only 3 of 28 patients with del(17p) (11%), suggesting that patients with t(4;14) show greater benefits with VRd + ASCT than patients with del(17p) [119]. Around 30% of patients with HRCAs who achieved MRD-negativity showed similar PFS compared with those who were MRD-negative and had standard-risk CAs, regardless of ASCT [119]. The IFM/DFCI study indicated that ASCT may abrogate the risk of HRCAs in a subpopulation.

In the phase 3 PETHEMA/GEM2012 study, 458 transplant-eligible NDMM patients, including 20% with HRCAs, treated with induction with 6 × VRd, the complete response (CR) rate at the end of induction was about 35% in both standard- and high-risk CA groups. However, the progressive disease rates during induction, which usually show dismal outcomes, were 20%, 13%, and 12% in patients with del(17p), t(4;14), and t(14;16), respectively, while it was 4% in patients with standard-risk CAs [120]. This indicates that the incidence of patients who are refractory to VRd is higher in those with HRCAs than in those with standard CAs.

The phase 2 GRIFFIN study compared Dara-VRd + ASCT vs. VRd + ASCT in transplant-eligible NDMM [121] (Table 2). Patients were first assigned to 4x (Dara-VRd) + ASCT + 2x (Dara-VRd) or 4 × VRd + ASCT + 2 × VRd, and subsequently assigned to maintenance with 26 × Dara-lenalidomide or 26 × lenalidomide. Dara-VRd improved the response rates and depth of responses, including MRD-negativity (10^–5^) rates at the end of consolidation (MRD-negativity rates, 55% vs. 25%, *p *< 0.0001). A subgroup analysis of MRD negativity (10^–5^) favored Dara-VRd in all prognostic subgroups, but was not statistically significant for patients with ISS stage 3 or HRCAs, which might be due to the small number of high-risk patients.

The phase 3 CASSIOPEIA study compared Dara-VTd (bortezomib, thalidomide, and dexamethasone) + ASCT to VTd + ASCT in transplant-eligible NDMM [113, 114] (Table 2). Patients were first assigned to 4x (Dara-VTd) + ASCT + 2x (Dara-VTd) or 4 × VTd + ASCT + 2 × VTd, and subsequently assigned to 2-year Dara (once every 8 weeks) or observation. The primary endpoint of part 1 of the study (induction to end of consolidation), stringent CR (sCR) assessed 100 days after transplantation, was better in the Dara-VTd group than in the VTd group (odds ratio 1.6, 95% CI 1.2–2.1, *p *= 0.0010), but the benefit of Dara-VTd was not seen in patients with HRCAs (sCR rate in the HRCAs group, Dara-VTd vs. VTd 24% vs. 28%, odds ratio 0.83, 95% CI 0.42–1.66). In contrast, regarding MRD-negativity (10^–5^) rates at day100 ASCT in patients with HRCAs, that in the Dara-VTd group showed better than that in the VTd group [(Dara-VTd vs. VTd 59% vs. 44%, odds ratio 1.8 (1.02–3.4)]. The GRIFFIN and CASSIOPEIA studies indicate that the addition of Dara to the induction and consolidation of ASCT can help reach deeper response, including MRD-negativity (10^–5^), which may lead to longer PFS in patients with HRCAs.

The phase 2 FORTE study compared the efficacy of KRd + ASCT vs. KRd alone vs. KCd + ASCT in transplant-eligible NDMM patients [122] (Table 2). Patients were first assigned to three groups: 4 × KRd + ASCT + 4 × KRd, 12 × KRd alone, and 4 × KCd + ASCT + 4 × KCd, and subsequently assigned to two groups of post-ASCT therapy, carfilzomib + lenalidomide (KR) or lenalidomide until disease progression. PFS in the KRd + ASCT group was superior compared with KRd alone and KCd + ASCT (vs. KRd alone, hazard ratio 0.48, 95% CI: 0.27–0.86; vs. KCd + ASCT, hazard ratio 0.34, 95% CI 0.15–0.77). KR as a maintenance therapy also improved PFS compared with lenalidomide alone (time to progression (TTP) from the second randomization, hazard ratio 0.63, 95% CI 0.43–0.92). The subgroup analyses showed a consistent benefit of KRd + ASCT + KRd as induction-intensification-consolidation and KR as maintenance in all prognostic subgroups, with similar hazard ratios among patients with standard-risk and high-risk CAs. The FORTE study indicated that ASCT has an important role for improving PFS in patients with HRCAs in the treatment with KRd. Maintenance with KR also appears to improve outcomes in patients with HRCAs, compared to lenalidomide alone.

**Fig. 2:**
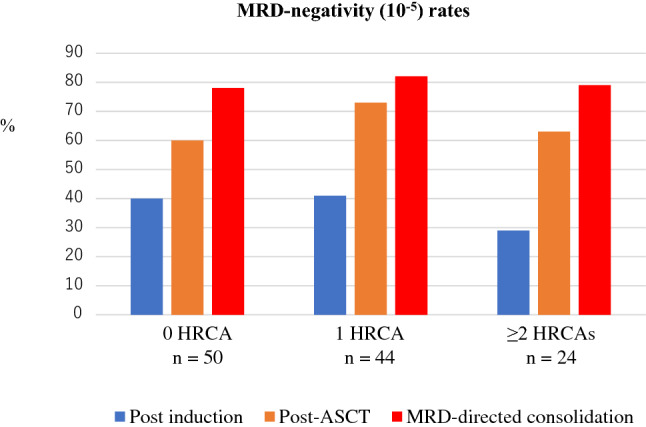
Proportion of achievement of MRD-negativity (MRD, 10^–5^) by treatment phase and the number of HRCA in the MASTER trial. The negativity rates increased from post induction to consolidation in all subgroups [45]. *MRD* minimal residual disease, *HRCA* high-risk cytogenetic abnormality, *ASCT* autologous stem cell transplantation

The single-arm, phase 2 MASTER trial treated transplant-eligible patients with 4 × Dara-KRd followed by ASCT and MRD-guided post-ASCT consolidation or cessation of therapy [45] (Table 2). Treatment cessation was done in patients with two consecutive MRD-negative assessments. MRD was evaluated at the end of induction, post-ASCT, and every four cycles (maximum of eight cycles) of consolidation with Dara-KRd. The trial enrolled 123 patients with NDMM with planned enrichment for HRCAs (the rate of patients with the number of HRCA: 0, 1, ≥ 2; 43%, 37%, and 20%, respectively). HRCAs included t(4;14), t(14;16), t(14;20), 1q21 + , and del(17p). The primary endpoint, achievement of MRD negativity (10^–5^), was achieved in 80% of patients (78%, 82%, and 79% for patients with 0, 1, and ≥ 2 HRCA, respectively) (Fig. 2), and 71% reached two consecutive MRD-negativity during therapy, entering treatment-free surveillance phase. Two-year PFS was 87% (91%, 97%, and 58% for patients with 0, 1, and ≥ 2 HRCA, respectively). The cumulative incidence of MRD resurgence or progression 12 months after cessation of therapy was 4%, 0%, and 27% for patients with 0, 1, or ≥ 2 HRCA, respectively. The MASTER trial indicates that Dara-KRd + ASCT, and MRD-guided consolidation can lead to a high rate of achievement of MRD-negativity in NDMM patients with 0 or 1 HRCA, but progression risk during or shortly after therapy is greatly increased in patients with ≥ 2 HRCAs. Patients with ≥ 2 HRCAs do not seem to be overcome even with the quartet of Dara-KRd with ASCT, so they need an alternative and novel treatment approach.

The phase 2 OPTIMUM trial treated transplant-eligible high-risk NDMM patients with an induction of 6 × Dara-C (cyclophosphamide) VRd and high-dose melphalan augmented with bortezomib, with ASCT followed by 18 × Dara-VR(d) consolidation and Dara-R maintenance [123] (Table 2). Results from 107 patients with ultra-high-risk (UHiR) by the trial definition (≥ 2 high-risk cytogenetics: t(4;14), t(14;16), t(14;20), 1q21 + , del(1p), del(17p)), or SKY92 high-risk, or with PCL, have been reported [123]. Among MRD evaluable patients, the MRD-negativity (< 10^–5^) rates at post-induction and day 100 post-ASCT were around 50% and 80%, respectively. In addition, a subpopulation progressed early in this highly intensive treatment setting.

In summary, ASCT seems to play an important role in improving responses, including MRD-negativity, in patients with HRCAs, which may lead to the improvement of PFS. Dara-KRd + ASCT, and MRD-adapted Dara-KRd consolidation can lead to high rates of sustained MRD-negativity in NDMM patients with 0 or 1 HRCA, but not for those with ≥ 2 HRCAs. The dismal prognostic effects of ≥ 2 HRCAs in NDMM patients cannot be fully abrogated by treatment with Dara-CVRd + ASCT, + Dara-VR(d) consolidation + Dara-R maintenance. These data suggest that there are still need for innovative novel treatment approach for NDMM patients with UHiR, including ≥ 2 HRCAs.

Novel immunotherapies such as CAR-T cells may improve outcomes for NDMM patients with HRCAs. CAR-T cells targeting BCMA (B-cell maturation antigen) have demonstrated substantial efficacy in highly refractory patients with MM [124]. CAR-T cells are currently being evaluated in earlier-line trials with great hope of achieving long-lasting remission of MM, including for high-risk patients [124]. Other novel immunotherapies such as bispecific T-cell engager (BiTE) antibodies and antibody drug conjugates (ADC), which can be available for more patients, are also being evaluated in earlier-line trials for patients with high-risk MM [125].

## Conclusion

Treatment outcomes of myeloma have improved greatly; however, a subpopulation of MM patients still experience early death due to disease progression. FISH cytogenetics are useful in predicting the prognosis of MM patients. In general, t(4;14), t(14;16), t(14;20), 1q21 + , del (1p), and del(17p) are considered HRCAs, and the co-existence of HRCAs results in even worse prognosis. Based on sub-analysis data of high-risk patients from pivotal clinical trials, extended intensive treatment, i.e., induction with a 3- or 4-drug regimen (PI/IMiD/Dex or PI/IMiD/Dex/an anti-CD38 antibody), high-dose chemotherapy with ASCT, and post-ASCT therapy with PI/IMiD appears to abrogate the dismal prognostic effects of HRCAs in subpopulations. In addition, it is strongly suggested that sustained MRD-negativity is a surrogate for longer survival, regardless of cytogenetic risk. Ongoing clinical trials for populations enriched with high-risk patients, using MRD as an endpoint, will reveal the efficacy of investigated treatments in the near future. In addition, despite post-relapse treatment for high-risk patients usually being difficult, BCMA-CAR-T cells are highly effective in subpopulations of RRMM, even with HRCAs. The introduction of CAR-T cells and other novel immunotherapies such as BiTE and ADC into earlier-lines might help overcome the dismal prognosis of high-risk MM. In addition to modifying available treatments and introducing novel immunotherapies, the development of targeted therapeutics based on molecular pathology associated with aggressive disease and/or resistance may also help to improve the outcomes of patients with high-risk MM.
